# Robust calling performance in frogs infected by a deadly fungal pathogen

**DOI:** 10.1002/ece3.2256

**Published:** 2016-07-27

**Authors:** Sasha E. Greenspan, Elizabeth A. Roznik, Lin Schwarzkopf, Ross A. Alford, David A. Pike

**Affiliations:** ^1^ College of Marine and Environmental SciencesJames Cook UniversityTownsvilleQueensland4811Australia; ^2^ Present address: Department of Integrative BiologyUniversity of South FloridaTampaFlorida33620

**Keywords:** Amphibian, *Batrachochytrium dendrobatidis*, body condition, calling effort, chytridiomycosis, disease, frog calling, life‐history trade‐offs, mate attraction, sublethal effects, vocalization

## Abstract

Reproduction is an energetically costly behavior for many organisms, including species with mating systems in which males call to attract females. In these species, calling males can often attract more females by displaying more often, with higher intensity, or at certain frequencies. Male frogs attract females almost exclusively by calling, and we know little about how pathogens, including the globally devastating fungus, *Batrachochytrium dendrobatidis,* influence calling effort and call traits. A previous study demonstrated that the nightly probability of calling by male treefrogs, *Litoria rheocola,* is elevated when they are in good body condition and are infected by *B. dendrobatidis*. This suggests that infections may cause males to increase their present investment in mate attraction to compensate for potential decreases in future reproduction. However, if infection by *B. dendrobatidis* decreases the attractiveness of their calls, infected males might experience decreased reproductive success despite increases in calling effort. We examined whether calls emitted by *L. rheocola* infected by *B. dendrobatidis* differed from those of uninfected individuals in duration, pulse rate, dominant frequency, call rate, or intercall interval, the attributes commonly linked to mate choice. We found no effects of fungal infection status or infection intensity on any call attribute. Our results indicate that infected males produce calls similar in all the qualities we measured to those of uninfected males. It is therefore likely that the calls of infected and uninfected males should be equally attractive to females. The increased nightly probability of calling previously demonstrated for infected males in good condition may therefore lead to greater reproductive success than that of uninfected males. This could reduce the effectiveness of natural selection for resistance to infection, but could increase the effectiveness of selection for infection tolerance, the ability to limit the harm caused by infection, such as reductions in body condition.

## Introduction

Animals maximize their fitness by adjusting their allocation of resources throughout their lifetimes (Stearns 1992; Roff 2002). Reproduction is an energetically costly behavior for many species. Pathogen infections can thus alter reproductive investment in complex ways. Individuals carrying infections may reduce their present investment in reproduction so that they can divert energy to manage the costs of infection, for example, by producing and increasing immune responses (Cade 1984; Bonneaud et al. 2003; Martin et al. 2003; Madelaire et al. 2013). For instance, male field crickets, *Gryllus integer*, call less frequently when parasitized by *Euphasiopteryx ochracea* flies (Cade 1984), and male Burmeister's treefrogs, *Hypsiboas prasinus*, decrease their calling rate with increasing helminth parasite intensity (Madelaire et al. 2013). Alternatively, infected individuals may increase their investment in reproduction to compensate for a potentially shortened life span (Agnew et al. 2000). For example, male *Drosophila nigrospracula* court more when parasitized by mites (*Macrocheles subbadius*; Polak and Starmer 1998).

Because male courtship behaviors are usually subject to sexual selection by females, male reproductive success is contingent not only on the quantity or frequency of reproductive behavior but also on its quality, which is evaluated by females during mate choice (Welch et al. 1998). In frogs, male advertisement calls are energetically intensive, and the spectral and temporal properties of calls that are associated with female mate choice are quantifiable, making them suitable for studies of disease‐mediated changes in courtship behavior (Blair 1964; Gerhardt and Doherty 1988; Cocroft and Ryan 1995; Welch et al. 1998; Hoskin et al. 2005; An and Waldman 2016). Female frogs generally prefer frequent, long, rapidly repeated, and low‐frequency calls (Gerhardt and Doherty 1988; Felton et al. 2006; Parris et al. 2009). These attributes often reflect high investment in calling, good body condition, or large body size, all of which may be directly important or may be honest indicators of male quality (Gerhardt and Doherty 1988; Felton et al. 2006; Parris et al. 2009). Parasite‐mediated models of sexual selection predict that the calls of unhealthy males will be less attractive than those of healthy males (Hamilton and Zuk 1982; Møller 1990). Females thus should prefer to mate with healthy males, thereby avoiding passing on genes for parasite susceptibility to their offspring. In contrast, if the sexual signals of unhealthy males are either indistinguishable from or more attractive than those of healthy males, females may pass on genes for disease susceptibility to their offspring (Pfennig and Tinsley 2002; An and Waldman 2016).

The amphibian pathogen *Batrachochytrium dendrobatidis* (hereafter Bd) has recently become endemic in many anuran populations worldwide (Farrer et al. 2011). We understand very little about how this pathogen influences frog behavior, including energetically costly mate attraction and reproductive behavior. If the calls of infected males are as or more attractive to females than those of uninfected males, as is hypothesized in Bd‐infected *Hyla japonica* (An and Waldman 2016), then these males could perpetuate infections by passing them to mates and by passing on genetic traits that render offspring susceptible to disease.

Our objective was to determine whether Bd infection alters individual call attributes commonly linked to mate choice. As a model species, we used a stream‐breeding rainforest frog from tropical Australia (*Litoria rheocola*) that breeds year‐round and is already known to exhibit a body condition‐dependent response of one aspect of male reproductive behavior (probability of calling each night) to Bd infection (Roznik et al. 2015a). In uninfected males of this species, the probability of calling each night (i.e., whether or not a frog calls on a given night) is independent of body condition (Roznik et al. 2015a). However, when males are infected by Bd, the probability of calling each night is significantly related to body condition. Specifically, infected frogs in the poorest body condition are 40% less likely to call than uninfected frogs, whereas those in the best body condition are 30% more likely to call than uninfected frogs (Roznik et al. 2015a).

The ultimate effect of these relationships on male reproductive success in *L. rheocola* is unknown, because Roznik et al. (2015a) did not examine the *characteristics* of the calls emitted by each individual. Males could modulate their call attributes according to their infection status or intensity in several possible ways: infected males could (1) enhance the quality of calls, for example, by calling more rapidly, producing longer calls, or producing calls at frequencies that are more attractive to females, as occurs in the seasonal‐breeding *Hyla japonica* (An and Waldman 2016). However, infected males (even those in good body condition) might be unable to expend additional energy to do this, and instead (2) might not adjust their call characteristics or (3) might even produce calls of lower quality, for example, by reducing their calling rate or the lengths of individual calls. We examined these possibilities using field‐collected recordings of natural calling by infected and uninfected males.

## Materials and Methods

### Study species and site

The common mistfrog, *L. rheocola*, is an Endangered (IUCN 2015) treefrog that occurs in association with rocky, fast‐flowing rainforest streams in northeastern Queensland, Australia (Roznik and Alford 2015). Males call year‐round, typically perching on streamside rocks and vegetation (Roznik et al. 2015a). By the mid‐1990s, chytridiomycosis (i.e., the disease caused by Bd) outbreaks had extirpated this species from high‐elevation portions of its range (>400 m a.s.l.; Richards et al. 1993; McDonald and Alford 1999). Many populations have subsequently recovered or recolonized in these areas and currently persist with endemic‐phase infections (McDonald et al. 2005; Phillott et al. 2013; Sapsford et al. 2013, 2015). We studied attributes of male advertisement calls in a recovered or recolonized population with endemic disease at Windin Creek in Wooroonooran National Park (750 m a.s.l.; 17.365°S, 145.717°E). This shallow, fast‐flowing stream varies in width (5–10 m), substrate size (small pebbles to large boulders), and structure (contains pools, runs, riffles, and waterfalls) and runs through dense tropical rainforest with abundant large trees (10 m in height), vines, epiphytes, shrubs, and herbaceous plants (Roznik et al. 2015b).

### Field sampling

We surveyed 61 calling male frogs along an 800‐m stream transect on 16 nights from June through September 2014. We recorded each calling male by positioning a tripod‐mounted directional microphone (ME 66, Sennheiser Nordic, Helsinki, Finland) and recorder (TASCAM DR‐2J, TEAC America Inc., Montebello, CA) approximately 1 m from the frog for 15 min. Audio waveforms were sampled at 48 kHz with 16‐bits per sample. To minimize disturbance, we used red lamps while positioning equipment and moved >10 m away from the frog while recording.

After each recording was complete, we measured the frog's body temperature using a noncontact infrared thermometer (OS425‐LS, Omega Engineering Ltd, Irlam, Manchester, U.K.; factory calibrated and accurate to ±1.0°C; emissivity 0.95; Rowley and Alford 2007). We then captured the frog and measured its body (snout‐urostyle) length (to 1 mm) and body mass (to 0.01 g). To determine infection status (infected or uninfected) and intensity (zoospore genome equivalents), we swabbed the ventral surface and limbs twice with a sterile rayon swab, which was then processed using diagnostic real‐time quantitative PCR (details below). To avoid re‐sampling the same individuals, we sampled a different section of stream each night. Individuals of this species have high site fidelity and are clustered in sections of the stream with riffles (Roznik and Alford 2015), making it highly unlikely that any individual was sampled more than once.

Fieldwork was carried out under Queensland Department of Environment and Heritage Protection permit WITK14585514 and James Cook University Animal Ethics permit A2023.

### DNA extraction and PCR

DNA was extracted from swabs using a modified Chelex ^®^ extraction protocol (Walsh et al. 1991). Individual swabs were placed into separate 1.5‐mL microcentrifuge tubes containing 3 *μ*L of proteinase K and 200 *μ*L of 5% Chelex^®^ solution. Samples were incubated at 55°C for 60 min and then at 95°C for 15 min with periodic vortexing. Extractions were stored at −20°C until required. Prior to PCR amplification, extractions were centrifuged at 12,000 *g* for 2 min and 2 *μ*L of supernatant from just above the Chelex^®^ resin was used for PCR amplification.

The real‐time quantitative PCR protocol was modified from Boyle et al. (2004). Assays were conducted using a Roche LightCycler 480 system in a 384‐well format. Ten *μ*L of reactions containing 5 *μ*L of 2× Qiagen multiplex PCR Master Mix (Qiagen, Valencia, CA), PCR primers at a concentration of 900 nmol/L, the MGB probe at 250 nmol/L, and 2 *μ*L of DNA were prepared in triplicate. Included in each assay plate were control reactions containing DNA from 40, 4, 0.4, and 0.04 Bd zoospore genome equivalents and controls with no DNA template. Standards were provided by the Australian Animal Health Laboratory, CSIRO. Amplification conditions were 15 min at 95°C followed by 15 sec at 95°C and 1 min at 60°C for 50 cycles. Amplification profiles of each PCR were used to determine the crossing point (Cp) value using the absolute quantification module of the LightCycler^®^ 480 software package. A standard curve was constructed from the control reactions containing 40, 4, 0.4, and 0.04 Bd zoospore genome equivalents, and this was used to determine the concentration of each sample, expressed as the number of zoospore genome equivalents (ZGE).

### Call attributes and statistical analysis

We measured call attributes using Raven Pro 1.5 software (Bioacoustics Research Program, Cornell Lab of Ornithology, Ithaca, NY). To avoid measuring call attributes at times when frogs may have been disturbed by investigators, we removed the first 4 min and the last 1 min of each 15‐min recording. This produced a 10‐min call sample, in which we measured attributes of the first call in each minute of the 10 min. Call attributes included duration (length of the call from beginning of the first pulse to end of the last pulse), pulse rate (number of pulses per second during call), dominant frequency (frequency that occurs at the highest amplitude), call rate (total number of calls in the 10‐min call sample), and intercall interval (length of time from end of the last pulse to beginning of the first pulse in the subsequent call). These attributes are routinely used in studies of communication and female mate choice in anurans (Blair 1964; Cocroft and Ryan 1995; Hoskin et al. 2005). We generated spectrograms with the Hamming window, 1024‐point discrete Fourier transformation, and 50% overlap.

Five of the sampled frogs each had one or two intercall intervals that exceeded 10 sec. We interpreted these values as outliers caused by momentary disturbances and excluded them from analyses. Prior to analyses, we log_10_‐transformed intercall interval values and infection intensity values. To obtain call attribute data for each frog for use in statistical analysis, we calculated the mean of the 8–10 measurements for each call attribute for each frog. We then modeled the effects of body temperature and body (snout‐urostyle) length on each call attribute and extracted residuals from the best supported model for each attribute (ΔAIC_c_ < 2). All subsequent analyses incorporated the extracted residuals, which we used as adjusted call attributes to investigate effects of infection, size‐independent body condition, and infection intensity. Size‐independent body condition was estimated as the residuals from a linear regression of log_10_‐transformed body mass on square‐root‐transformed body length (Peig and Green 2009).

We performed a principal component analysis (PCA) to visualize the relationships among the corrected call attributes and to suggest any differences in call attributes that might exist between infected and uninfected frogs. We tested for the effects of infection status and size‐independent body condition on our adjusted call attributes using multivariate analysis of covariance (MANCOVA), with infection status as a classification variable and the estimate of each frog's body condition as the covariate. To determine whether infection intensity might affect call attributes, we performed a separate canonical correlation analysis using data for infected frogs only, with adjusted call attributes as the dependent variables and log_10_‐transformed infection intensity and body condition as independent variables. We used R software for all statistical analyses (version 3.0.3; R Core Team 2014).

## Results

Thirty‐six of the 61 frogs that we recorded (59%) were infected by Bd (mean infection intensity ± SD = 22.99 ± 74.49 zoospore equivalents; maximum infection intensity = 426.7 zoospore equivalents). Overall, mating calls consisted of a series of pulses lasting 0.56 to 1.20 sec (mean ± SD = 0.87 ± 0.14 sec) that were emitted at a rate of 45–76 pulses per s (mean ± SD = 57.82 ± 7.75 pulses per sec) and a dominant frequency of 2.33–3.08 kHz (mean ± SD = 2.64 ± 0.17 kHz; Table 1).

**Table 1:** Call attributes, body temperatures, body sizes, and body condition indexes of common mistfrogs, *Litoria rheocola*, with and without infections by the fungus *Batrachochytrium dendrobatidis*

	Infected *n* = 36			Uninfected *n* = 25			Overall *n* = 61
	Minimum	Mean ± SD	Maximum	Minimum	Mean ± SD	Maximum	Mean ± SD
Duration (sec)	0.56	0.85 ± 0.14	1.20	0.58	0.89 ± 0.14	1.20	0.87 ± 0.14
Pulse rate (pulses/sec)	44.98	57.56 ± 7.82	76.28	46.80	58.20 ± 7.79	73.52	57.82 ± 7.75
Dominant frequency (kHz)	2.34	2.65 ± 0.18	3.08	2.33	2.61 ± 0.16	2.99	2.64 ± 0.17
Call rate (calls/10 m)	93	257.6 ± 67.3	423	65	238 ± 74.4	350	249.5 ± 70.3
Intercall interval (sec)	0.89	1.57 ± 0.62	4.09	0.99	1.67 ± 0.79	4.08	1.61 ± 0.69
Body temperature (°C)	11.40	15.10 ± 1.80	18.90	11.10	15.40 ± 1.90	18.50	15.01 ± 1.83
Body (snout‐urostyle) length (mm)	28	30.17 ± 1.16	33	29	30.32 ± 0.96	33	30.23 ± 1.07
Mass (g)	1.63	1.98 ± 0.19	2.38	1.78	2.03 ± 0.13	2.25	2.00 ± 0.17
Body condition index	−0.39	−0.01 ± 0.19	0.46	−0.24	0.01 ± 0.15	0.41	0.00 ± 0.17

**Figure 1:**
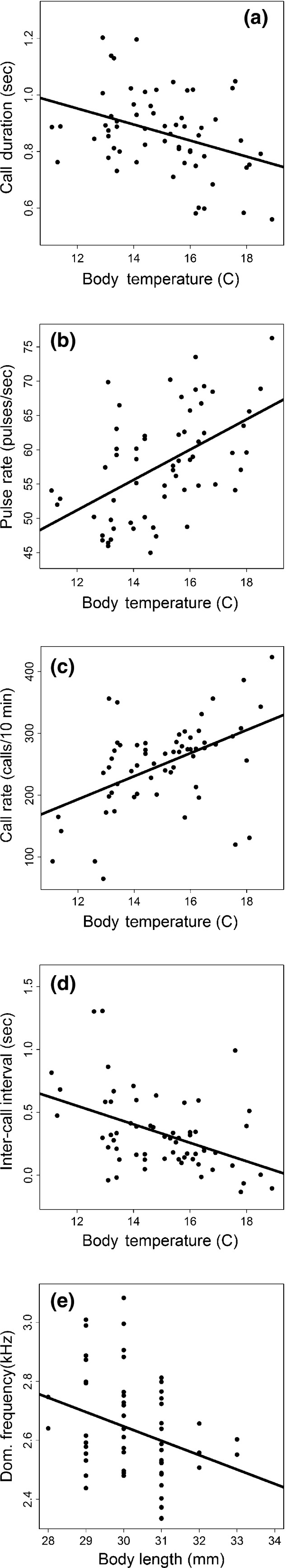
Relationships between frog body temperature or frog body (snout‐urostyle) length and advertisement call attributes for the common mistfrog, *Litoria rheocola*. Intercall interval values were log_10_‐transformed.

Model selection indicated that both body temperature and body length influenced call attributes (Table 2, Fig. 1) and that call duration, pulse rate, call rate, and intercall interval were largely mediated by temperature (Fig. 1A–D). Call duration decreased and pulse rate increased with increasing body temperature (Fig. 1A and B). Similarly, call rate increased and intercall interval decreased with increasing body temperature (Fig. 1C and D). Dominant frequency was inversely correlated with body length (Fig. 1E).

**Table 2:** Candidate models of the effects of frog body temperature and body (snout‐urostyle) length on the advertisement call attributes of common mistfrogs, *Litoria rheocola*. Models with ΔAIC_c_ < 2 are bolded

Response	Predictors	AIC_c_	ΔAIC_c_	Weight	Adjusted *R* ^2^
**Duration**	**Temperature**	−**67.3**	**0.00**	**0.710**	**0.000**
	Temperature, Length	−65.2	2.12	0.245	0.000
	Intercept only	−60.9	6.43	0.028	0.000
	Length	−59.8	7.51	0.017	0.000
**Pulse rate**	**Temperature**	**409.0**	**0.00**	**0.636**	**0.273**
	**Temperature, Length**	**410.1**	**1.12**	**0.363**	**0.286**
	Intercept only	426.2	17.17	0.000	0.000
	Length	428.4	19.37	0.000	0.000
**Dominant frequency**	**Length**	**800.8**	**0.00**	**0.567**	**0.092**
	**Temperature, Length**	**802.3**	**1.54**	**0.263**	**0.103**
	Intercept only	804.4	3.67	0.091	0.000
	Temperature	804.7	3.95	0.079	0.031
**Call rate**	**Temperature**	**681.0**	**0.00**	**0.730**	**0.236**
	**Temperature, Length**	**683.0**	**1.99**	**0.269**	**0.240**
	Intercept only	695.2	14.22	0.001	0.000
	Length	697.2	16.22	0.000	0.003
**Intercall interval**	**Temperature**	**20.3**	**0.00**	**0.756**	**0.543**
	Temperature, Length	22.6	2.29	0.240	0.543
	Intercept only	31.4	11.04	0.003	0.000
	Length	32.9	12.56	0.001	0.031

**Figure 2:**
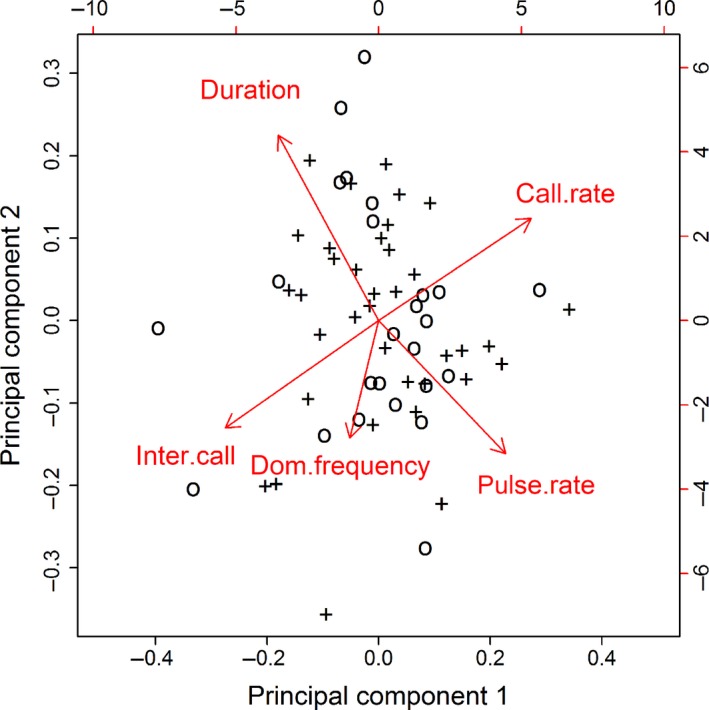
Biplot showing the relationships between adjusted advertisement call attributes of common mistfrogs, *Litoria rheocola*, and illustrating the location of each individual data point in principal component space. We adjusted the data by removing the effects of frog body temperature and body (snout‐urostyle) length from the data for each frog. Principal components 1 and 2 accounted for 70% of the variability in call attribute data (Table 3). The length and direction of each arrow correspond to the loading of each adjusted call attribute on the first two principal axes (Table 4). Symbols indicate frog infection status (infected [+] or uninfected [o]). Interspersion of the symbols suggests that infected and uninfected frogs do not differ in call attributes commonly linked to mate choice.

After we adjusted the calling parameters for significant effects of temperature and body length, our principal component analysis of the adjusted call attributes indicated that the first two principal components together accounted for 71% of the variability in the data (Table 3). The analysis revealed that the temporal attributes of calls – call duration, pulse rate, call rate, and intercall interval – were still correlated (Table 3, Fig. 2). The inverse correlations between call duration and pulse rate and the complex relationships between call rate, call duration, and intercall interval make it appear that frogs alter their calls by adjusting the proportion of energetic input that lies along dimensions roughly corresponding to rate (faster pulse and/or faster emission of entire calls) and duration (length of each call, length of time between calls; Fig. 2). In particular, it appears that higher pulse rates are balanced against shorter calls and that longer calls are produced by decreasing the pulse rate, maintaining a relatively constant number of pulses (Fig. 2). Higher call rate is produced by decreasing intercall interval. Even with the effect of body length removed, dominant frequency varies relatively independently of the other call characteristics (Fig. 2).

**Table 3:** Summary of the importance of components and axis loadings in a principal component analysis of adjusted advertisement call attributes of common mistfrogs, *Litoria rheocola*. We adjusted the data by removing the effects of frog body temperature and body (snout‐urostyle) length from the data for each frog

	PC1	PC2	PC3	PC4	PC5
Standard deviation	1.522	1.121	0.969	0.620	0.325
Proportion of variance	0.463	0.251	0.188	0.077	0.021
Cumulative proportion	0.463	0.714	0.902	0.979	1.000
Loading values					
Duration	−0.370	0.625	0.161	0.659	0.111
Intercall interval	−0.565	−0.362	−0.208	−0.042	0.710
Pulse rate	0.466	−0.449	−0.217	0.721	0.122
Dominant frequency	−0.106	−0.397	0.906	0.098	−0.016
Call rate	0.561	0.345	0.249	−0.189	0.684

We found no significant difference in adjusted call characteristics between infected and uninfected animals; in the PCA, both the distributions of individual points and the locations of group centroids were quite similar (Fig. 2). This was confirmed by the results of the MANCOVA and the canonical correlation analysis (Table 4). After accounting for the effects of body temperature and body length on the attributes of frog calls, we found no significant or substantial effect of infection status, infection intensity, or body condition (Table 4, Fig. 2).

**Table 4:** Summary of multivariate analysis of covariance and canonical correlation analysis of the effects of *Batrachochytrium dendrobatidis* infections on adjusted advertisement call attributes of common mistfrogs, *Litoria rheocola*. We adjusted the data by removing the effects of frog body temperature and body (snout‐urostyle) length from the data for each frog. The adjusted call attributes included in each analysis as dependent variables were call duration, pulse rate, dominant frequency, call rate, and intercall interval

Main effect	Predictors	Wilk's *λ*	df	*F*	*P*
Infection status	Infection status	0.907	5,53	1.092	0.376
	Body condition	0.994	5,53	0.068	0.997
	Infection status × body condition	0.952	5,53	0.531	0.752
Infection intensity	Infection intensity	0.814	5,28	1.278	0.301
	Body condition	0.945	5,28	0.327	0.892
	Infection intensity × body condition	0.763	5,28	1.744	0.157

## Discussion

Body temperature explained much of the observed variation in call duration, pulse rate, call rate, and intercall interval, independent of frog infection status. We observed a general trend of increasing pulse rate and decreasing call duration with increasing frog body temperatures (Fig. 1A and B). Similarly, we observed an increase in call rate and a decrease in intercall interval with increasing frog body temperatures (Fig. 1C and D). Physiological rates, including those associated with vocalizations (Zweifel 1968; Gayou 1984; Narins et al. 2006), are positively correlated with temperature in anurans because they are ectotherms (Feder 1992). It follows that call duration is typically negatively correlated with temperature (Zweifel 1968; Gayou 1984; Narins et al. 2006) because a call will be shorter if a given number of pulses are closer together in time (Gayou 1984). In the same way, the interval between calls will be shorter if there are more calls in a given length of time. The inverse relationship between the dominant frequency of calls and frog body length (Fig. 1E) was expected given that body size is the primary determinant of dominant frequency (Zweifel 1968; Martin 1971; Davies and Halliday 1978; Robertson 1986).

After accounting for the effects of temperature and body length on call characteristics, we found no significant influence of Bd infection status or intensity on the call duration, pulse rate, dominant frequency, call rate, or intercall interval of the rainforest frog, *Litoria rheocola*. These results are similar to findings that dominant frequency and call duration were not related to helminth parasite load in the treefrogs *Hyla versicolor* (Hausfater et al. 1990) and *Hypsiboas prasinus* (Madelaire et al. 2013), but differ from recent findings in Bd‐infected *Hyla japonica*, which called more rapidly and had longer calls than uninfected frogs (An and Waldman 2016), suggesting that behavioral responses of frogs to infection by Bd differ among species. Overall, our results suggest that for *L. rheocola*, (1) lightly infected males produce calls with sounds that should be, on average, as attractive to females as the calls of uninfected males (see duration, pulse rate and dominant frequency in Tables 1) and (2) on the nights that they call, lightly infected males produce, on average, equal numbers of calls compared to uninfected males (see call rate and intercall interval in Table 1).

Because none of the standard aspects of call quality differed between infected and uninfected males, the changes that Bd infection produces in the nightly probability of calling of *L. rheocola* (i.e., more likely to call on a given night when in good condition and less likely to call on a given night when in poor condition; Roznik et al. 2015a) should lead to changes in mating opportunities. Our results, together with those reported by Roznik et al. (2015a), thus suggest that infected males in poor body condition are likely to mate less often than uninfected males, while infected males in good body condition should mate more often than uninfected males. Whether this pattern leads to changes in the success of matings, and ultimately in offspring production, may depend on how Bd infection affects other aspects of male and female reproductive behavior and physiology (e.g., effects of infection on gametogenesis; Brannelly et al. 2015).

How infection alters male calling effort clearly differs among species and may depend on species or environmental context, such as susceptibility to Bd and the duration of the breeding season. An and Waldman (2016) recently documented increases in three of ten call quality parameters tested in nine male *H. japonica* lightly infected (15–43 zoospore equivalents) with Bd (compared to 33 uninfected individuals). *H. japonica* is not known to suffer morbidity and mortality from this pathogen, unlike our species *L. rheocola*, which is susceptible to Bd and has undergone population declines, but is currently persisting with endemic infections. The costs of fighting infection may therefore be greater for *L. rheocola* than for *H. japonica*, leaving less energy available to *L. rheocola* for increasing call quality. In addition, *H. japonica* breeds during a distinct summer period, which gives males a smaller temporal window to successfully call and attract mates. By contrast, our tropical species breeds throughout the year, which provides males with a much longer temporal window during which they must balance call quality with reproductive endurance (Roznik et al. 2015a), and in which calling on more nights is likely to lead to increased opportunities for mating.

Increases in recruitment, which could reflect either increased survival to adulthood or increased reproduction, have been proposed as a population‐regulating process in Bd‐infected populations of *L. rheocola* (Phillott et al. 2013; Sapsford et al. 2015), *L. verreauxii alpina* (Scheele et al. 2015), and *Anaxyrus boreas* (Muths et al. 2011) with low adult survival. Our results, combined with those of Roznik et al. (2015a) demonstrate that infected male *L. rheocola* in good body condition call on more nights, emitting calls that should be as attractive to females as those of uninfected males. Whether this results in compensatory increases in recruitment is uncertain; it would only do so if male reproductive effort is limiting. If male reproductive effort is not limiting, however, there may be no effect, or a negative effect caused by increased transmission of Bd infections to females during amplexus (Rachowicz and Vredenburg 2004).

Although models of parasite‐mediated sexual selection suggest that females should discriminate against infected males based on changes in their courtship signals or behavior, this may not be possible for female *L. rheocola*, as infected male *L. rheocola* did not alter any of the measured aspects of the quality of their advertisement calls. Based only on the characteristics of mating calls, we would expect that the reproductive success of resistant males that remain uninfected and susceptible males that are infected should be similar, thus reducing the effects of natural selection for resistance to infection in this system. In this species, however, the probability of calling on any given night depends on both infection status and body condition (Roznik et al. 2015a). Infected males in relatively good body condition are more likely to call each night than are uninfected males, while both these classes of individuals are more likely to call each night than are infected males in poor condition (Roznik et al. 2015a). It is thus likely that infected males in good body condition have greater success in attracting mates than do uninfected males because they call on more nights. Any genes that favor increased tolerance of infection (i.e., ability to limit the harm caused by infection, such as reductions in body condition) may thus be favored by natural selection in this system. The presence of a behavioral strategy for maximizing individual male reproductive success in the face of infection may thus have altered the course of natural selection following the emergence of chytridiomycosis in *L. rheocola*. Similar effects may make the evolutionary responses of other species to the emergence of novel diseases difficult to predict.

## Conflict of Interest

None declared.
